# Role of Oral Intake, Mobility, and Activity Measures in Informing Discharge Recommendations for Hospitalized Inmate and Noninmate Patients With COVID-19: Retrospective Analysis

**DOI:** 10.2196/43250

**Published:** 2023-06-27

**Authors:** Matthew S Briggs, Erin Shevawn Kolbus, Kevin Michael Patterson, Lindsay Elizabeth Harmon-Matthews, Shana McGrath, Catherine C Quatman-Yates, Cristiane Meirelles, Marka Jean Salsberry

**Affiliations:** 1 Rehabilitation Services The Ohio State University Wexner Medical Center Jameson Crane Sports Medicine Institute Columbus, OH United States; 2 Sports Medicine Research Institute The Ohio State University Wexner Medical Center Columbus, OH United States; 3 Department of Orthopaedics The Ohio State University Wexner Medical Center Columbus, OH United States; 4 School of Health and Rehabilitation Sciences, College of Medicine The Ohio State University Columbus, OH United States

**Keywords:** incarceration, Functional Oral Intake, Activity Measure for Postacute Care, speech language pathology, physical therapy, occupational therapy, COVID-19

## Abstract

**Background:**

Patients who were incarcerated were disproportionately affected by COVID-19 compared with the general public. Furthermore, the impact of multidisciplinary rehabilitation assessments and interventions on the outcomes of patients admitted to the hospital with COVID-19 is limited.

**Objective:**

We aimed to compare the functional outcomes of oral intake, mobility, and activity between inmates and noninmates diagnosed with COVID-19 and examine the relationships among these functional measures and discharge destination.

**Methods:**

A retrospective analysis was performed on patients admitted to the hospital for COVID-19 at a large academic medical center. Scores on functional measures including the Functional Oral Intake Scale and Activity Measure for Postacute Care (AM-PAC) were collected and compared between inmates and noninmates. Binary logistic regression models were used to evaluate the odds of whether patients were discharged to the same place they were admitted from and whether patients were being discharged with a total oral diet with no restrictions. Independent variables were considered significant if the 95% CIs of the odds ratios (ORs) did not include 1.0.

**Results:**

A total of 83 patients (inmates: n=38; noninmates: n=45) were included in the final analysis. There were no differences between inmates and noninmates in the initial (*P*=.39) and final Functional Oral Intake Scale scores (*P*=.35) or in the initial (*P*=.06 and *P*=.46), final (*P*=.43 and *P*=.79), or change scores (*P*=.97 and *P*=.45) on the AM-PAC mobility and activity subscales, respectively. When examining separate regression models using AM-PAC mobility or AM-PAC activity scores as independent variables, greater age upon admission decreased the odds (OR 0.922, 95% CI 0.875-0.972 and OR 0.918, 95% CI 0.871-0.968) of patients being discharged with a total oral diet with no restrictions. The following factors increased the odds of patients being discharged to the same place they were admitted from: being an inmate (OR 5.285, 95% CI 1.334-20.931 and OR 6.083, 95% CI 1.548-23.912), “Other” race (OR 7.596, 95% CI 1.203-47.968 and OR 8.515, 95% CI 1.311-55.291), and female sex (OR 4.671, 95% CI 1.086-20.092 and OR 4.977, 95% CI 1.146-21.615).

**Conclusions:**

The results of this study provide an opportunity to learn how functional measures may be used to better understand discharge outcomes in both inmate and noninmate patients admitted to the hospital with COVID-19 during the initial period of the pandemic.

## Introduction

### Background

Discharge destination is often used as an outcome metric for hospitalized patients [1-3]. To optimize care strategies, it is important to understand the factors that may influence or predict this outcome, particularly for those with COVID-19 [1-3]. As multidisciplinary rehabilitation approaches facilitate improved functional status for hospitalized patients, it is important to understand how the combination of functional measures, such as oral intake, mobility, and activity measures, is related to discharge destination [4-7]. A better understanding of these factors could help optimize rehabilitation interventions and outcomes (including discharge destination) in hospitalized patients with COVID-19 and other acute respiratory diseases [7].

For example, deficiencies in functional oral intake, as measured by the Functional Oral Intake Scale (FOIS), are prevalent (42%-61%) in individuals following mechanical ventilation and result in an increased risk of poor outcomes [8-11]. In addition, functional status as measured by the Activity Measure for Postacute Care (AM-PAC) mobility and activity scores have been shown to be independent predictors of outcomes in individuals hospitalized with and without COVID-19, including discharge destination, mortality, and length of hospital stay [2,3]. However, the impact of using a combination of these measures (eg, FOIS and AM-PAC) in predicting the discharge destination is unknown.

Furthermore, it is important to understand how outcomes may vary in different patient populations with COVID-19. Patients who were incarcerated were disproportionately affected by COVID-19 compared with the general public [12-15]. More specifically, prisoners demonstrated a more severe presentation of disease characteristics and had worse outcomes (eg, higher intensive care unit [ICU] admissions, higher hospital mortality rate, and higher 30-day mortality rate) than nonincarcerated patients [13-15]. Constrained mobility, confined and overcrowded spaces, limited access to resources, and high prevalence of mental health disorders contribute to increased risk of individuals who are incarcerated acquiring transmissible diseases such as COVID-19 [14,16-18]. Furthermore, approximately 16% (male) and 10% (female) of prisoners in federal and state prisons were aged 50 years in 2021 [19]. As many incarcerated individuals aged ≥55 years have chronic conditions, such as heart and lung conditions, this puts them at an even greater health risk [18,20,21]. Given the greater disease risk and burden of COVID-19 in those who were incarcerated, it is important to understand whether rehabilitation assessments and interventions have similar impacts in those who are and are not incarcerated. However, the impact of rehabilitative care and related outcomes for prisoners with COVID-19 has not been reported. Considering the disproportionate impact of COVID-19 on those who were incarcerated, it is important to understand the functional outcomes in this population [12-15].

### Purpose

The first purpose of this study was to compare the functional outcomes (FOIS and AM-PAC scores) between inmates and noninmates who were diagnosed with COVID-19 and received rehabilitation services (eg, speech, physical, and occupational therapy) while admitted to an inpatient hospital in the initial months of the COVID-19 pandemic. The second purpose of this study was to examine the relationships among FOIS scores, AM-PAC scores, and discharge destination in this same sample of patients given the interdisciplinary nature of rehabilitation care. A better understanding of these results, particularly during the initial phase of the COVID-19 pandemic, may inform care plan development, including discharge planning, to maximize outcomes in patients with COVID-19 and other acute respiratory diseases. Our first hypothesis was that there would be no difference in the FOIS and AM-PAC scores between inmates and noninmates. Our second hypothesis was that initial AM-PAC and FOIS scores would predict whether patients were discharged on a total oral diet with no restrictions (FOIS score=7). Our third hypothesis was that the initial AM-PAC and FOIS scores would predict whether patients were discharged to the same destination as they were admitted from.

## Methods

### Study Design and Setting

A retrospective analysis was performed on patients admitted to the hospital for COVID-19 at a large academic medical center between February 2020 and August 2020. Data were obtained from the academic medical center. The hospital where the data collection occurred was the primary referral source of the state correctional facilities in the region and disproportionately saw the majority of inmates with COVID-19 compared with other hospitals in the region. Furthermore, the medical center was a transfer facility for all patients requiring an escalation of medical interventions. The patients’ care in this study was based on medical necessity and was not based on incarceration status.

### Ethics Approval

The study was approved by The Ohio State University’s institutional review board (protocol #2020H0367), as well as the State Department of Rehabilitation and Corrections.

### Data Collection

The inclusion criteria for this study were as follows: (1) patients who were deemed COVID-19 positive and admitted to the medical center between February 2020 and August 2020, (2) those who had both baseline and discharge FOIS scores (meaning they were referred for a swallowing evaluation), and (3) those who had at least a baseline and discharge AM-PAC (mobility and activity) score. Patients were included if they were admitted with a COVID-19 diagnosis or were found to have a COVID-19 diagnosis during their admission to the hospital for another reason. The exclusion criteria were as follows: (1) patients who were not diagnosed with COVID-19 at the medical center or (2) those with COVID-19 who died during the hospital stay or were placed on comfort care or hospice as either they did not have a living discharge destination or their diets were often adjusted for comfort care, thus impacting their final FOIS score.

Patient and clinical data were obtained via a manual chart review. Data obtained included inmate status (yes or no); admission and discharge dates and admission source (eg, home, skilled nursing facility, other hospital, or correctional facility); discharge destination and sex (male or female); hospital length of stay; intubation status (yes or no); days requiring mechanical ventilation and baseline and discharge FOIS score; baseline and discharge AM-PAC scores (mobility and activity subscales); height, weight, and BMI upon admission; age upon admission; and race.

### Variables

The dependent variables included (1) whether patients achieved an FOIS score of 7 at discharge from the hospital and (2) whether patients were discharged to the same destination as they were admitted from. Initially, admission and discharge destinations were categorized as (1) home, (2) correctional facility, (3) outside the hospital, (4) skilled nursing facility, (5) extended care facility, (6) long-term acute care hospital, and (7) inpatient rehabilitation facility. Discharge destination was then dichotomized to either “discharge to same destination of admittance” or “discharge to different destination than admittance.” If a patient was discharged to the same destination from which they were admitted (eg, home or correctional facility), this was considered a positive outcome. However, if a patient was discharged to a different type of facility, location, or institution than that they were admitted from (eg, admitted from home but discharged to a skilled nursing facility), this was considered an inferior outcome based on the need of higher care intensity. There were two exceptions to this coding: (1) if a patient was admitted from an extended care facility but discharged to a skilled nursing facility or vice versa (n=4) or (2) if an inmate patient was admitted from an outside hospital but discharged to a correctional facility (n=5). These 2 exceptions were considered better outcomes and the data were coded as “discharge to same destination source.” As we were interested in better understanding how intake or baseline information may be used to prognosticate outcomes in patients with COVID-19, the initial FOIS and AM-PAC (mobility and activity subscales) scores were the primary independent variables of interest.

An initial bedside swallowing evaluation was performed by a speech language pathologist when the patients were deemed medically stable and appropriate by the ordering provider and speech language pathologist. Being medically stable was determined on a patient-by-patient basis and was fundamentally based on the patients’ vital signs stabilizing or not degrading. If patients were on a ventilator, they would need to be off the ventilator before the FOIS could be administered. An order for a swallowing evaluation may have occurred in the ICU or outside the ICU (eg, step-down unit). The FOIS was used to rate a patient’s functional oral intake during the swallowing evaluation. These evaluations occurred on average 13 to 14 days following hospital admission for noninmates and inmates, respectively. There was no difference between noninmates and inmates with regard to when the FOIS was administered. The FOIS scores were based on clinical bedside swallowing evaluation. The FOIS is a commonly used tool and has excellent agreement (85%-95%) and excellent interrater reliability (*k*=0.86-0.91) [22,23]. The FOIS has also been shown to have strong consensual validity (W=0.90) [22]. FOIS scores are used to categorize (levels 1-7) and document clinical changes in oral intake of food and liquids in patients with dysphagia. Levels 1 to 3 relate to varying degrees of tube-dependent or nonoral feeding, and levels 4 to 7 relate to varying degrees of oral feeding without feeding tube use or nonoral supplementation [22,23]. Levels 4 to 6 relate to both diet modifications and patient compensations, whereas a level 7 relates to a total oral diet with no restrictions [22,23].

The AM-PAC short-form measure “6-Clicks” was administered to patients by a physical therapist (mobility subscale) and an occupational therapist (activities of daily living subscale) during their respective initial evaluations and subsequent treatment sessions [24]. The referral for physical or occupational therapy was made based on the physician team’s determination that the patient would benefit from physical or occupational therapy interventions. This referral may have occurred in the ICU or outside the ICU. The administration of the AM-PAC could occur while the patient was on a ventilator. On average, the AM-PAC was administered between 8 and 9 days following hospital admission for noninmates and inmates, respectively. There was no difference between noninmates and inmates with regard to when the AM-PAC was administered. The AM-PAC has two scales that are used to assess patient physical function: (1) basic mobility (eg, walking and moving positions) and (2) activities of daily living (eg, dressing and toileting) [24,25]. The AM-PAC “6 Clicks” has been validated in the acute care setting and has good overall reliability for the basic mobility (intraclass correlation coefficient=0.849) and daily activity (intraclass correlation coefficient=0.783) subscales [24,25]. Patient status on the AM-PAC scales is based on assistance needed on a scale of 1 (“total”) to 4 (“none”) with 6 mobility- and daily living–related activities [24,25]. For each scale, values are summed and raw scores are standardized, with higher scores indicating higher levels of function [24,25].

Other variables that were included as covariates were nonmodifiable demographic characteristics that have been shown to impact outcomes in patients with COVID-19 [2,26-30]. These variables included race, age, and sex. Race was categorized as “White,” “Black,” or “Other” [2,27]. The “Other” category was created owing to the limited numbers of patients who did not fit the racial categories of “White” or “Black.” In addition, this category also included patients who “refused to answer” or “did not know.” Sex was categorized as “male” or “female” [28,30]. Age was used as a continuous variable [26,29,30]. In addition, as incarceration status has been associated with worse outcomes in patients with COVID-19, this variable was also included [12-15].

### Statistical Analysis

**Figure 1:**
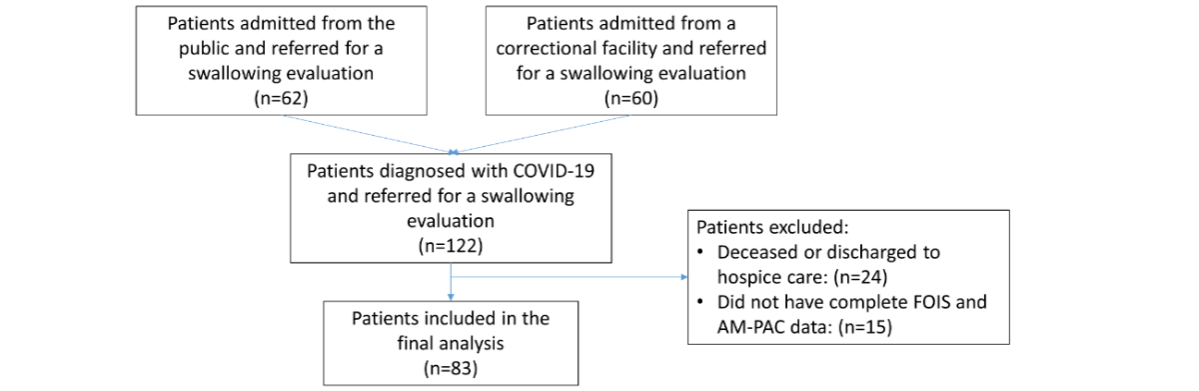
A flowchart outlining patients who were included in the analysis. A total of 62 patients from the public and 60 patients from correctional facilities were admitted to the hospital and were referred for a swallow evaluation as well as physical therapy and occupational therapy. A total of 24 patients were excluded secondary to being deceased or discharged to a hospice care. Furthermore, 15 patients were excluded secondary to not having a complete data set of Functional Oral Intake Scale (FOIS) or Activity Measure for Postacute Care (AM-PAC) data. A total of 83 patients (38 inmates and 45 noninmates) were included in the analysis.

The data obtained were deidentified. The flow diagram in Figure 1 illustrates the inclusion and exclusion of the obtained data. A total of 62 patients from the public and 60 patients from correctional facilities were admitted to the hospital and were referred for a swallow evaluation as well as physical therapy and occupational therapy. A total of 24 patients were excluded secondary to being deceased or discharged to a hospice care. Furthermore, 15 patients were excluded secondary to not having a complete data set of FOIS or AM-PAC data. A total of 83 patients (38 inmates and 45 noninmates) were included in the analysis. In addition, the baseline FOIS scores were initially categorized as 1 to 7 (nothing by mouth through total oral diet without restrictions); however, owing to the lack of variability in each of the categories, the baseline FOIS scores were ultimately categorized on the basis of whether the patients were on a total oral diet with (FOIS score=1-6) or no restrictions (FOIS score=7).

Descriptive and frequency statistics were used to characterize the sample. Shapiro-Wilk tests were used to determine whether the variables were normally distributed. Mann-Whitney *U* tests were used to compare AM-PAC scores between inmates and noninmates. Chi-square tests were used to compare the distributions of categorical variables between inmates and noninmates. Wilcoxon signed-rank tests were used to compare pre-post AM-PAC scores in inmates and noninmates separately to examine the change in scores between baseline and final measurements. Significance was set at *P*<.05 for any comparison tests. For the second purpose of this study, it was determined a priori that if there was no difference between the inmate and noninmate groups based on the results of the Mann-Whitney *U* tests among the primary variables of interest, then the data would be pooled for the logistic regression analysis. As there was no difference between the groups, binary logistic regression was performed on the entire data set to evaluate the relationships between (1) the identified variables and achieving a total oral diet with no restrictions (FOIS score=7) and (2) whether functional scores (FOIS and AM-PAC scores) would predict whether patients were discharged to the same destination as they were admitted. AM-PAC mobility scores and AM-PAC activity scores were determined to be multicollinear. Thus, separate logistic regressions were conducted, one with AM-PAC mobility scores as an independent variable and the other with AM-PAC activity scores as an independent variable. The goodness of fit of the model to the data was evaluated using the Omnibus test of the model, the Hosmer and Lemeshow test, and Nagelkerke *R*^2^ value. The independent variables were entered into the model together using the “enter” method. All assumptions of logistic regression were met for the final models. There was no evidence of multicollinearity (based on tolerance, 0.786-0.945, and variance inflation factor, 1.058-1.272 statistics) among the independent variables. Furthermore, the independent continuous variables were linearly related to the log odds, as determined by the Box-Tidwell test. Independent variables were considered statistically significant if the 95% CIs of the odds ratios (ORs) did not include 1.0. Statistical analyses were performed using SPSS software (version 28; IBM Corp).

## Results

### Demographics

Overall, 83 patients were included in the final sample, with an average age of 62 (SD 13.31) years. Most of the patients were male (61/83, 73%), White (46/83, 54%), and intubated at least 1 time (59/83, 71%), and approximately half (27/59, 46%) of those who were intubated were inmates. There were no differences in age (*P*=.38), length of hospital stay (*P*=.42), length of intubation (*P*=.37), or BMI (*P*=.90) between the inmates (n=35) and noninmates (n=45; Table 1).

There were differences between inmate and noninmate patients in terms of race distribution (*P*<.001; Table 2). Furthermore, there was no difference in terms of intubation (*P*=.99), being “discharged to the same admittance source” (*P*=.13), or being discharged with an FOIS score of 7 (*P*=.18; Table 2).

**Table 1:** Continuous demographics (n=83).

Characteristics	Inmates (n=38), mean (SD)	Noninmates (n=45), mean (SD)
Age at admission (years)	61.3 (10.2)	63.4 (15.5)
Length of hospital stay (days)	24.9 (12.0)	24.8 (15.4)
Length of intubation (days; if intubated)	14.0 (6.1)	13.0 (8.2)
Height at admission (cm)	178.4 (10.2)	167.4 (9.7)
Weight at admission (kg)	100.9 (30.9)	88.3 (24.8)
BMI at admission (kg/m^2^)	31.6 (8.3)	31.3 (7.7)

**Table 2:** Categorical demographics (n=83).

Descriptors		Inmates (n=38), n (%)	Noninmates (n=45), n (%)
**Race**			
	White	21 (55)	25 (56)
	Black	17 (45)	6 (13)
	Other	0 (0)	14 (31)
**Sex**			
	Male	38 (100)	23 (51)
	Female	0 (0)	22 (49)
**Intubation**			
	Yes	27 (71)	32 (71)
	No	11 (29)	13 (29)
**Discharged to the same admittance source**			
	Yes	28 (74)	26 (58)
	No	10 (26)	19 (42)

### FOIS and AM-PAC Scores Between Inmate and Noninmate Patients

The majority of patients (inmates: 24/38, 63%; noninmates: 29/45, 64%) had a baseline FOIS score of 1 (nothing by mouth) while the majority (inmates: 25/38, 66%; noninmates: 23/45, 51%) also had a discharge FOIS score of 7 (total oral diet with no restrictions; Table 3).

Although both groups demonstrated improvement in their AM-PAC mobility and activity scores when compared within each group (*P*<.001), there were no significant differences between inmates and noninmates at the initial, final, or change scores (all *P*>.05; Table 4).

**Table 3:** Functional Oral Intake Scale (FOIS) scores (n=83).

FOIS scores^a^			Inmates, n (%)		Noninmates, n (%)
**Initial**					
	1	24 (63)		29 (64)	
	4	1 (3)		0 (0)	
	5	11 (29)		10 (22)	
	7	2 (5)		6 (13)	
**Discharge**					
	1	1 (3)		3 (7)	
	5	12 (32)		19 (42)	
	7	25 (66)		23 (51)	

^a^A comparison of the distribution of initial FOIS score (*P*=.39) and distribution discharge FOIS score (*P*=.27) between inmates and noninmates.

**Table 4:** A comparison of Activity Measure for Postacute Care (AM-PAC) score measures between inmates and noninmates (n=83).

AM-PAC score^a^			Inmates (n=38), mean (SD)		Noninmates (n=45), mean (SD)		*P* value
**Basic mobility AM-PAC**							
	Initial score	10.4 (4.3)		9.0 (4.6)		.06	
	Final score	14.5 (5.0)		13.6 (5.8)		.43	
	Change score	4.0 (4.6)		4.6 (6.2)		.97	
**Daily activity AM-PAC**							
	Initial score	11.6 (4.6)		10.8 (4.5)		.46	
	Final score	14.2 (4.7)		14.4 (4.8)		.79	
	Change score	2.6 (5.3)		3.6 (5.9)		.45	

^a^Comparisons using the Mann-Whitney *U* test between inmates and noninmates.

### Predictors of Being Discharged With Total Oral Diet With No Restrictions

Results from the logistic regression models examining the odds of achieving an FOIS score of 7 at discharge demonstrated that greater age upon admission to the hospital decreased the odds of a patient being discharged with an FOIS score of 7 (total oral diet with no restrictions). This was true for both regression models using the independent variable AM-PAC mobility scale (OR 0.922, 95% CI 0.875-0.972) or the independent variable AM-PAC activity scale (OR 0.918, 95% CI 0.871-0.968; Table 5). Inmate status, race, sex, baseline AM-PAC mobility score or activity score, and baseline FOIS score were not significant variables within the regression models (Table 5).

**Table 5:** Odds of being discharged from the hospital with a Functional Oral Intake Scale score of 7.

Predictors				Odds ratio (95% CI)
**Model 1^a^**				
	Inmate status (yes)			1.04 (0.255-4.248)
	**Race**			
		White (reference)	—^b^	
		Black	0.973 (0.289-3.277)	
		Other	0.195 (0.035-1100)	
	Female sex			1.171 (0.285-4.819)
	Age on date of hospital admission (years)^c^			0.922 (0.875-972)
	**Baseline mobility AM-PAC^d,e^ score**			1.12 (0.987-1.284)
		Baseline FOIS^f^ score	1.094 (0.854-1.401)	
**Model 2^g^**				
	Inmate status (yes)			1.327 (0.340-5.180)
	**Race**			
		White (reference)	—	
		Black	0.942 (0.278-3.188)	
		Other	0.249 (0.048-1.287)	
	Female sex			1.36 (0.339-5.459)
	Age on date of hospital admission (years)^h^			0.918 (0.871-0.968)
	**Baseline activity AM-PAC score^e^**			1.062 (0.947-1.191)
		Baseline FOIS score	1.149 (0.906-1.458)	

^a^Nagelkerke *R*^2^=0.298; Hosmer and Lemeshow Test: *P*=.96.

^b^No data as the independent variable “White” is used as the reference variable for the other categorical variables (“Black” and “Other”) in the regression model.

^c^Overall model: *P*=.004.

^d^AM-PAC: Activity Measure for Postacute Care.

^e^Baseline basic mobility AM-PAC and baseline basic activity AM-PAC scores highlight the different independent variables included in each of the models.

^f^FOIS: Functional Oral Intake Scale.

^g^Nagelkerke *R*^2^=0.268; Hosmer and Lemeshow Test: *P*=.08.

^h^Overall model: *P*=.01.

### Predictors of Being Discharged to the Same Admittance Source

When examining the logistic regression results related to discharge destination, inmate status increased the odds (OR 5.285, 95% CI 1.334-20.931 and OR 6.083, 95% CI 1.548-23.912) that a patient was to be discharged to the same destination as where they were admitted from in both models (Table 6). Moreover, being categorized as “Other” race increased the odds on a magnitude of 7 to 8.56 times (Table 6) when using those who were “White” as the reference group. Being female also increased the odds of being discharged to the same place of admission (OR 4.671, 95% CI 1.086-20.092 and OR 4.977, 95% CI 1.146-21.615). Age, AM-PAC scores, and FOIS scores were not significant variables in the regression models examining the discharge destination (Table 6).

**Table 6:** Odds of being discharged from the hospital to the same admission source.

Predictors				Odds ratio (95% CI)
**Model 1^a^**				
	Inmate status (yes)^b^		5.285 (1.334-20.931)	
	**Race**			
		White (reference)	—^c^	
		Black	1.837 (0.529-6.375)	
		Other^b^	7.596 (1.203-47.968)	
	Female sex^b^		4.671 (1.086-20.092)	
	Age on date of hospital admission (years)		1.009 (0.964-1.055)	
	**Baseline mobility AM-PAC^d^ score^e^**		1.13 (0.973-1.313)	
		Baseline FOIS^f^ score	1.018 (0.796-1.303)	
**Model 2^g^**				
	Inmate status (yes)^h^		6.083 (1.548-23.912)	
	**Race**			
		White (reference)	—	
		Black	1.961 (0.557-6.908)	
		Other^h^	8.515 (1.311-55.291)	
	Female sex^h^		4.977 (1.146-21.615)	
	Age on date of hospital admission (years)		1.003 (0.959-1.050)	
	**Baseline activity AM-PAC score^e^**		1.109 (0.973-1.264)	
		Baseline FOIS score	1.042 (0.820-1.324)	

^a^Nagelkerke *R*^2^=0.230; Hosmer and Lemeshow Test: *P*=.82.

^b^Overall model: *P*=.03.

^c^No data as the independent variable “White” is used as the reference variable for the other categorical variables (“Black” and “Other”) in the regression model.

^d^AM-PAC: Activity Measure for Postacute Care.

^e^Baseline basic mobility AM-PAC and baseline basic activity AM-PAC scores highlight the different independent variables included in each of the models.

^f^FOIS: Functional Oral Intake Scale.

^g^Nagelkerke *R*^2^=0.226; Hosmer and Lemeshow Test: *P*=.53.

^h^Overall model: *P*=.04.

## Discussion

### Principal Findings

Patients who are incarcerated are a vulnerable population in health care systems, have been disproportionately affected by COVID-19, and have been shown to have worse health outcomes than the general population [12-15,31]. Some factors suggested to contribute to these findings may include an increased virus exposure and transmission risk owing to the potential for overcrowding and high contact with others as well as increased preexisting health conditions (eg, chronic obstructive pulmonary disease or cardiovascular disease) compared with nonincarcerated individuals [12-15,31]. However, in our study, there were no differences in health outcomes between inmates and noninmates related to the FOIS and AM-PAC. Functional scores from the FOIS and AM-PAC did not change the odds of being discharged with an FOIS score of 7; however, older age did decrease these odds. The FOIS and AM-PAC scores did not change the odds of patients being discharged to the same admission source. However, inmate status, “Other” race, and female sex did increase the odds of being discharged to the same admittance source. No prior studies have compared FOIS and AM-PAC scores between inmate and noninmate patients nor has the relationship between the use of AM-PAC scores and FOIS scores in patients hospitalized with COVID-19 been examined.

### FOIS and AM-PAC Scores Between Inmate and Noninmate Patients

This is the first study to compare functional measures using the FOIS and AM-PAC between inmate and noninmate patients with COVID-19 or otherwise. Previous literature during a similar period of the COVID-19 pandemic has demonstrated that inmate patients with COVID-19 have higher rates of ICU admissions, intubation, hospital mortality, and 30-day mortality rate and a higher incidence of acute kidney injury compared with noninmate patients with COVID-19 [13-15]. However, there was no difference in the FOIS and AM-PAC scores in our study when comparing inmates to noninmates. Furthermore, both inmate and noninmate patients demonstrated improvement in their initial FOIS and AM-PAC scores compared with the scores at discharge. This lack of difference in outcomes (based on the FOIS and AM-PAC) is contrary to previous literature, comparing inmates with noninmates [13-15]. However, the FOIS and AM-PAC measure different constructs of outcomes (eg, functional) than outcomes measured or reported comparing inmates with noninmates. These functional measures are likely one of many components of potential outcomes. This illustrates how these measures (FOIS and AM-PAC) may provide additional input regarding function over time and their potential relationship with other outcome measures and constructs in these populations.

### Predictors of Being Discharged With Total Oral Diet With No Restrictions

This is the first study to examine the predictors of being discharged with a total oral diet with no restrictions (eg, an FOIS score of 7) in patients with COVID-19. Previous literature examining functional oral intake in patients with COVID-19 admitted to the hospital demonstrated that FOIS scores are associated with the number of days in the hospital but improve from the initial assessment to discharge [32]. Our study supports these results, demonstrating an improvement from the initial to discharge FOIS scores. However, the initial functional measures of FOIS and AM-PAC (mobility and activity scales) scores did not contribute to either model regarding patients with COVID-19 being discharged from the hospital with a normal diet (FOIS score=7; Table 5). Greater age consistently decreased the odds of being discharged with a normal diet (FOIS score=7; Table 5). This is not surprising and supports previous literature demonstrating that older patients with COVID-19 are more likely to have worse outcomes [26,29,30].

### Predictors of Being Discharged to the Same Admittance Source

Function and mobility (using multiple measures) have been shown to be predictors of discharge destination in general medical and rehabilitation [1,24,33-38]. More specifically, Tevald et al [2] demonstrated that AM-PAC mobility and activity scores were independent predictors of discharge disposition in patients with COVID-19. However, our findings demonstrate contrary results that neither the AM-PAC mobility nor the activity scales changed the odds of discharge destination in those with COVID-19. Our study adds to these findings while also including functional oral intake as an additional measure of functional status in the logistic regression models. Although FOIS scores did not change the odds of discharge destination among the other variables, their inclusion did improve the overall regression model.

In addition, results from this preliminary investigation showed that inmate status was a strong predictor in our regression models of whether patients were discharged to their same admittance source (or better discharge destination outcome). The results demonstrate that being an inmate increased the odds on a magnitude of 5 to 6 times of being discharged to the same admittance source (Table 6). Including inmate status as an independent variable in the regression analysis improved model fit. These results should be interpreted with caution, as inmates were more likely to have fewer choices of discharge destinations than the general population. However, inmates would have to reach a level of stability and health before being able to be discharged back to their respective correctional facility. Other options for discharge destination for inmates included a correctional facility hospital that provided less medical care than the academic medical center but more care than their respective correctional facility. This illustrates that it may be important to consider whether patients are inmates when examining outcomes, especially as it relates to discharge destination. However, this also requires further investigation to clarify the potential differences in outcomes between inmates and noninmates.

In addition, race also affected the discharge destination. When using “White” as the reference category, “Other” races increased the odds of patients being discharged to the same admission source between 7 and 8.5 times (Table 6), thus suggesting lower care needs or intensity. This is a notable finding, considering that racial and ethnic minority individuals with COVID-19 have been shown to have worse outcomes than non–racial and ethnic minority individuals and delayed access to services such as palliative care [2,27,39,40]. This may be because of the limited number of patients in this category. However, this finding is in contrast with other studies examining relationships among race and discharge destination in other patient populations [39,41]. For example, being “Black” or “Asian” has been shown to be associated with being discharged to an extended care facility versus home in patients following total hip arthroplasties [39,41]. It has been suggested that discharge destination is determined not only by clinical parameters but also by other social determinants of health that may be associated with race, such as home proximity, social and community support, and other markers of social deprivation [39,41-43]. Thus, the opposite phenomenon identified in our results, at least pertaining to “Other” races, requires further investigation to better understand the relationship of race and discharge destination in those with COVID-19.

In addition, being female increased the odds (4-5 times) of being discharged to the same source of admission. Lewis et al [44] demonstrated that female patients with COVID-19 were more often discharged home than to a rehabilitation facility when compared with male patients. Furthermore, male patients with COVID-19 have been shown to have worse health outcomes than female patients [45,46]. As such, female patients being more likely to be discharged to the same admission source may be reflective of better outcomes.

Finally, although greater age was an important factor related to being discharged with an FOIS score of 7 (Table 5), it was not an important factor related to discharge destination (Table 6). This is contrary to the findings of Lewis et al [44] who demonstrated that patients with COVID-19 that were older were more likely to be discharged to a rehabilitation facility than home. However, a systematic review examining factors predicting discharge destination in patients with stroke deemed age to be a “controversial” variable to consider related to discharge destination [47]. Furthermore, it is suggested that age may have a lower influence on discharge destination compared with other factors, such as function [47]. As our study was primarily focused on examining functional health outcomes (eg, FOIS and AM-PAC scores) among others, these factors may be overshadowing any effect age may have on the discharge destination.

### Limitations and Future Research

The results of this study have limitations that are worth considering. First, the data were obtained from a large academic medical center in a large midwestern metropolitan area. This may limit the generalizability of the results to other hospitals and geographic locations that were differently impacted by COVID-19 [48-53]. Furthermore, our data are focused on the first wave of the pandemic and a limited time frame when medical and rehabilitation providers were most challenged with identifying optimal management strategies for patients with COVID-19 and encountered different challenges (shortage of personal protective equipment, lack of vaccination availability, etc) [54-60]. At that time, clinical practice guidelines specific to the COVID-19 population were only emerging [61]. However, we felt it important to focus on this initial stage to reflect on and inform future rehabilitation management strategies for patients with COVID-19 or otherwise. In addition, our analysis was limited by the accuracy and consistency of the information entered into the electronic medical record. Errors and omissions by clinicians and others entering the data have the potential to influence the results. Furthermore, we did not categorize patients based on ICU admission. We felt it was important to include all patients based on the ultimate outcomes of them being discharged. However, in future, categorizing patients by ICU status may provide additional insights based on patient severity. In the regression analysis, there were a low number of patients who were categorized as “Other” for race and were not appropriate to be included in the other defined categories. Thus, the regression results should be interpreted with caution.

Opportunities for future research may include conducting a similar analysis during different phases of the pandemic to better understand how treatment strategies and other factors may impact results and how patient outcomes may change over the course of the pandemic for both inmate and noninmate populations. There is a need for descriptive and correlational studies to better understand the effects and outcomes of patients diagnosed with COVID-19 and the impact of rehabilitation services. Furthermore, as our results indicate that inmate status and race also influence discharge destination, factors related to incarceration, race, and discharge destination require clarification. In addition, examining the current functional status of this included cohort of patients may help provide important information regarding the long-term outcomes of patients with COVID-19 who require hospitalization.

### Conclusions

The results of this study provide an opportunity to learn how functional measures, such as the FOIS and AM-PAC, may be used to better understand discharge outcomes in both inmate and noninmate patients admitted to the hospital with COVID-19 during the initial period of the pandemic. Our preliminary findings provide input on how inmate status, age, race, and sex may impact the outcomes (eg, discharge destination and oral intake) examined in patients diagnosed with COVID-19. These factors require further clarification as they relate to outcomes in patients with COVID-19. Finally, additional investigation is necessary regarding the utility of the FOIS and AM-PAC scores in understanding patient outcomes in those with COVID-19.

## Abbreviations

AM-PAC: Activity Measure for Postacute Care
FOIS: Functional Oral Intake Scale
ICU: intensive care unit
OR: odds ratio
